# A Text Book of Opthalmogy

**Published:** 1893-11

**Authors:** Arthur G. Hobbs


A TEXT-BOOK OF OPHTHALMOLOGY. By William F. Norris, M. D., Professor of Ophthalmology in the University of Pennsylvania, and Charles A. Olliver, M. D., Surgeon to Wills Eye Hospital, Philadelphia. In one very handsome octavo volume of 641 pages, with 357 engravings and 5 colored plates. Cloth, $5.00; leather $6.00. Philadelphia; Lea Bros. & Co., 1893.

A new text book on Opthalmology, by Drs. Norris and Oliver has been recently issued in a beautiful style, by the Lea Brothers. It is in many respects the most complete and perfect treatise on Physiological Optics and on Eye Diseases that has been issued from any press during the last decade, if not the most perfect that has ever appeared.

The first part, by Dr. Oliver, deals particularly with the mathematical part of the subject much of which would seem yet to be in its chrysilis stage as regards its clinical applications if we judge by the numerous journal articles on this subject. For this reason this part of the book will be of most interest to the Opthalmologist, particularly at this time. The author has handled this difficult task well and by apt illustrations has succeeded in establishing order where chaos existed in the minds of many.

The second part of the book by Dr. Norris, is equally as well handled. It will prove of more interest to the general practitioner than to the oculist, but the latter will be well repaid for the time spent in closely reading it. He will not infrequently be reminded of modes for treatment that, in his routine work, may have escaped him. This occurs to most of us no doubt oftener than we care to acknowledge to ourselves.

All the more recent modes of treatment that have been based upon late physiological and pathological researches, particularly all that have stood the test, are tersely but quite amply enough described.

The chapter on retinal diseases is, I think, the best ever written on this subject in any text book in the language.

ARTHUR G. HOBBS.

